# Platelet‐to‐lymphocyte ratio a potential prognosticator in acute myocardial infarction: A prospective longitudinal study

**DOI:** 10.1002/clc.24002

**Published:** 2023-04-14

**Authors:** Hongling Wang, Li Li, Yi Ma

**Affiliations:** ^1^ Second Department of Cardiology Tangshan Gongren Hospital Tangshan Hebei China

**Keywords:** acute myocardial infarction, AMI, MACE, major adverse cardiac events, platelets to lymphocytes ratio, PLR, prognostic factors

## Abstract

**Background:**

The ratio of platelets to lymphocytes (PLR) can serve as a potential biomarker for predicting the prognosis of individuals with acute myocardial infarction (AMI).

**Aim:**

The purpose of the research was to evaluate the in‐hospital outcomes of AMI patients and the predictive significance of PLR on major adverse cardiac events (MACE).

**Methods:**

A total of 799 AMI patients who had successful primary PCI within 12 h of the onset of chest pain were separated into low PLR (*n* = 511) and high PLR (*n* = 288) groups using a PLR cutoff value of 178. At admission, total white blood cell, neutrophil, lymphocyte, and platelet counts were assessed.

**Results:**

In patients with a high PLR group with PLR > 178, the incidence of MACE: heart rupture, acute heart failure, total adverse events, and mortality due to all events was considerably greater. In an analysis of the receiver operating characteristic curve, a high PLR > 178 accurately predicted adverse outcomes (73% specificity and 65% sensitivity). Age, hypertension, and PLR were found as independent predictors of adverse outcomes by multiple logistic regression.

**Conclusions:**

AMI patients with high PLR had poor hospital outcomes. These findings recommend PLR as an independent risk factor for hospital‐acquired complications, suggesting that inflammation and prothrombotic state may contribute to the poor prognosis of high PLR patients.

## Highlights

- Numerous studies have revealed a strong correlation between platelets to lymphocytes (PLR), a possible indicator of inflammation, and coronary artery disease (CAD), and concluded that PLR might serve as a valid predictor of mortality or major adverse cardiac events in addition to CAD.
- However, whether PLR accurately predicts the prognostic outcome of patients with CAD is still controversial, and it still lacks knowledge about the connection between PLR and acute myocardial infarction (AMI).
- The findings of this study showed that the PLR might be regarded as a potentially accessible, dependable, and affordable criterion for risk stratification and categorization in AMI patient groups.

## Graphical abstract

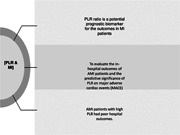

## 1 INTRODUCTION

Atherosclerosis is significantly linked to both leukocyte and platelet count.^1^ Myocardial cell necrosis 2 results from sudden full blockage of the artery lumen caused by rupture of atherosclerotic plaque in coronary arteries is the most common cause of acute myocardial infarction (AMI). AMI is the leading cause of death and morbidity from cardiovascular disease globally. Patients with AMI still have a poor prognosis and an inadequate survival rate, despite recent advances in reperfusion techniques.^2^, ^3^ Therefore, it is crucial to improve the treatment of AMI and patients' prognoses by identifying prognostic and diagnostic biomarkers for early risk assessment and in‐time therapeutic techniques. The relevance and importance of inflammatory processes in atherosclerosis have been shown by several research in both animals and humans.^4^, ^5^ Numerous studies have revealed a strong correlation between platelets to lymphocytes (PLR), a possible indicator of inflammation, and coronary artery disease (CAD), and concluded that PLR might serve as a valid predictor of mortality or major adverse cardiac events (MACEs) in addition to CAD. Similar to the potential marker NLR, neutrophil lymphocyte ratio, which reflects inflammation and the body's stress response via a decrease in the quantity of lymphocytes,^6^, ^7^, ^8^, ^9^, ^10^ PLR, platelet lymphocyte ratio, also indicates inflammation and the activity of thrombotic processes via platelet or lymphocyte counts.^8^, ^9^, ^11^, ^12^, ^13^ Previous studies have demonstrated that NLR could serve as an emerging biomarker of the incidence and severity of acute coronary syndrome (ACS).^14^, ^15^ It has been reported that high NLR may lead to the incidence of cardiac events or even cardiac death in AMI patients.^15^, ^16^, ^17^ However, whether PLR accurately predicts the prognostic outcome of patients with CAD is still controversial, and it still lacks knowledge about the connection between PLR and AMI. Many researchers have indicated that elevated NLR is connected with higher clinical adverse events, whereas the PLR did not show this association or there is little knowledge of the association of PLR with clinical features of AMI.^18^, ^19^ This study is going to investigate the connection between the PLR and 30‐day MACE with AMI patients following ST elevation myocardial infarction (STEMI) after they got successful PCI treatment.

## 2 OBJECTIVE

The purpose of the research was to evaluate the in‐hospital outcomes of AMI patients and the predictive significance of PLR on MACE.

## 3 EXPERIMENTAL

### 3.1 Study design

This research is a prospective longitudinal study conducted in Tangshan Tongren Hospital in Hebei, China, from January to December 2021. All experimental methods were authorized by the Ethics Committee of Tangshan Tongren Hospital in Hebei, China (ethics number: GRYY‐LL‐KJ2022‐002) and were conducted in accordance with the Declaration of Helsinki (2014).^20^

### 3.2 Setting

If the rise in troponin I is more than 1 ng/mL and J point is less than 2, the elevation of the new ST‐segment is measured. In accordance with standards published by the American Heart Association and European Heart Association, discomfort within the first 12 h was classified as AMI. Individuals with any blood issue, including anemia, any systemic illness, regardless of whether it is caused by inflammation or autoimmunity, any renal or hepatic disease, cancer, or use of NSAIDs or steroids in the last 6 months will not be included.

### 3.3 Participants

The research included AMI patients who had successful primary PCI within 12 h of the onset of chest symptoms. As the PLR cutoff value was 178, the patients (*n* = 799; male: 643, female: 156; mean age: 59.5−10.6 [SD] years) were divided into two groups. Participants signed the patient permission form after a thorough description of the projects' aims, advantages, and even potential risks.

### 3.4 Variables

Patients included in the research were questioned on the presence of hyperlipidemia, hypertension, diabetes, and smoking. Patients' age, sex, weight, and height were recorded simultaneously. Hyperlipidemia was diagnosed when total cholesterol was higher than 200 mg/dL, LDL was greater than 130 mg/dL, or triglycerides were greater than 150 mg/dL. Arterial hypertension was diagnosed when systolic or diastolic blood pressure exceeded 140 or 90 mmHg, respectively, more than twice, or when a history of antihypertensive drug use was present. Plasma glucose in the fasting state more than 126 mg/dL in any measurement, glycosylated hemoglobin fraction larger than 6.5%, or a history of using antidiabetic medication constituted hypertension. Current heavy cigarette consumers were smokers. On admission, we collect a blood sample from the patient and analyze it to determine the number of various cell types.

### 3.5 Data sources/measurement

#### 3.5.1 Coronary angiography

The Gensini score is examined by two interventional cardiologists who protect the confidentiality of clinical information throughout the coronary angiography evaluation. If the stenosis of the infarct artery is less than 30% and the thrombolytic therapy of the myocardial infarction is effective, we consider the operation a success. Before conducting the coronary intervention, the patient is given aspirin and loading dosages of clopidogrel or troglitazone (300 and 300/180 mg, respectively). The patient mandated to continue taking clopidogrel (75 mg/day) or troglitazone (90 mg/day) for more than 1 year and aspirin (100 mg/day) for life after the operation. Other drugs, including low molecular weight heparin, ACEI/ARBs, IIb/IIIa receptor antagonists, beta‐blockers, and nitrates, must be administered as prescribed.

#### 3.5.2 Quantitative variables

The clinical follow‐up data was obtained through medical case records or by calling patients and their family members. MACE, which consists of nonfatal myocardial reinfarction and cardiovascular mortality within 30 days of an AMI, has been designated as the research's primary outcome. Angiography was utilized to develop the nonfatal reinfarction of the myocardium. We establish the origin of the thrombus, whether it arises from the stent, the proximal or distal 5 mm of the stent, or both. Myocardial infarction is caused by both complete and partial blockage of the artery, accompanied by recurrence of chest discomfort and new ECG alterations in cardiac biomarkers. Cardiac events were identified as the cause of cardiovascular death.

#### 3.5.3 Statistical analysis

The graphical data were compared using the 22 *χ* ^2^ test, while the measurement data were analyzed using the two‐sample *t*‐test. Using ROC curves, the sensitivity and specificity of PLR and NLR for predicting 30‐day MACE were determined. Multivariate logistic models examining independent correlations between NLR or PLR and 30‐day MACE were adjusted for the following variables: age, sex, history of hyperlipidemia, hypertension, and diabetes, smoking, and BMI. Last but not least, Kaplan−Meier (KM) curves were created to quantify the cumulative risk of MACE 30 days after admission, and log‐rank tests were then performed to examine the predictive ability of NLR and PLR. SPSS 16.0 was used to conduct statistical analysis.

## 4 RESULTS AND DISCUSSION

### 4.1 Clinical outcomes

The PLR ratio is calculated by dividing the absolute platelet count by the absolute lymphocyte count. It is a novel inflammatory marker that has the potential to be employed in the prediction of inflammation and mortality in a variety of disorders. Table 1 outlines the MACE data. Patients with a high PLR had a higher incidence of nonfatal myocardial reinfarction (6.94% vs. 2.35%), cardiovascular death (8.33% vs. 2.35%), and MACE (15.28% vs. 4.70%) (*p* = .001). In the high PLR group, the occurrences of reinfarction, death, and MACE were approximately thrice higher than in the low PLR group.

**Table 1:** Comparison of major adverse cardiac events between of the study groups.

	Reinfarction *N* (%)	Cardiac death *N* (%)	MACE	*p* Value
PLR < 178	12 (2.35)	12 (2.35)	24 (4.70)	.012
PLR > 178	20 (6.94)	24 (8.33)	44 (15.28)	.001
*χ* ^2^	6.279	10.159	17.178	.000

Abbreviations: MACE, major adverse cardiac events; PLR, platelets to lymphocytes.

### 4.2 Baseline characteristics

Table 2 presents the features of baseline and relevant clinical data for all applicants. The average age in the high PLR group was 61 ± 10 years compared to 59 ± 11 years in the low PLR group (*p* = .001), and the proportion of smokers was 22.70% compared to 12.50% (*p* = .001). In both groups, the gender and prior medical history distribution of patients was almost identical. However, the BMI was greater in the group with a low PLR, 25.60 ± 3.18 against 23.89 ± 2.83 (*p* = .001). Blood parameters, such as white blood cell (WBC) count (11.19 ± 3.25) versus (10.57 ± 3.17) 10^9^/L, (*p* = .008), neutrophil count (9.74 ± 3.10) versus (8.25 ± 2.99) 10^9^/L, (*p* = .003), platelet count (253.24 ± 70.64) versus (207.35 ± 57.12) 10^9^/L, (*p* = .012), neutrophil to lymphocyte (11.73 ± 9.73 vs. 5.30 ± 2.70), (*p* = .002) were higher in the high PLR group, while lymphocyte count (0.97 ± 0.28 vs. 1.73 ± 0.64), (*p* = .007) was lower. According to coronary angiography, the number of infected arteries and Gensini score were comparable across groups, and the anterior site of myocardial infarction was more than 40% in all cases. Concerning in‐hospital medicine, the low PLR group is more likely to utilize—blockers (45.99% vs. 37.50%) and ACEIs/ARBs (35.81% vs. 26.74%), (*p* = .009).

**Table 2:** Demographic information, laboratory tests, and clinical characteristics of study groups.

Variables	Low PLR [control group] (*n* = 511)	High PLR (*n* = 288)	*t*/*χ* ^2^	*p*
Age (year)	59 ± 11	61 ± 10	2.614	.009
Risk factors				
Male	410 (80.23)	233 (81.02)	0.052	.819
Diabetes	136 (26.61)	60 (20.83)	3.325	.072
Hypertension	229 (44.81)	144 (50.00)	1.990	.158
Hyperlipidimia	51 (9.98)	20 (6.94)	2.197	.148
Smoking	116 (22.70)	36 (12.50)	12.441	.002
BMI	25.60 ± 3.18	23.89 ± 2.83	6.904	.015
Physical examination				
Systolic BP (mmHg)	124.65 ± 16.71	122.58 ± 14.62	1.816	.070
Diastolic BP (mmHg)	75.58 ± 10.04	74.15 ± 9.15	2.046	.041
Heart rate (beat/min)	78 ± 16	83 ± 18	3.756	.003
Laboratory finding				
WBC (10^9^/L)	10.57 ± 3.17	11.19 ± 3.25	2.668	.008
Neutrophil (10^9^/L)	8.25 ± 2.99	9.74 ± 3.10	6.752	.024
Lymphocyte (10^9^/L)	1.73 ± 0.64	0.97 ± 0.28	23.390	.002
NLR	5.30 ± 2.70	11.73 ± 9.73	11.062	.017
Platelet (10^9^/L)	207.35 ± 57.12	253.24 ± 70.64	9.417	.023
PLR	126.22 ± 33.10	298.74 ± 23.36	12.531	.007
Coronary angiographic finding				
Infarct‐related artery				
LAD	180 (46.88)	93 (43.05)	0.813	.367
LCX	62 (16.14)	19 (8.80)	6.394	.011
RA	142 (36.98)	104 (48.15)	7.129	.008
Number of diseased arteries	1.9 ± 0.7	1.8 ± 0.8	1.114	.266
Gensini score	62.96 ± 27.10	64.21 ± 26.70	0.544	.587
Pain to balloon (min)	4.73 ± 2.52	5.60 ± 3.07	3.905	.002
Medication				
Aspirin	507 (99.22)	283 (98.26)	1.503	.220
Clopidogrel/tricagrelor	507 (99.22)	283 (98.26)	1.503	.220
Statins	488 (95.50)	279 (96.88)	0.907	.341
β‐blockers	235 (45.99)	108 (37.50)	5.417	.020
ACEIs/ARBs	183 (35.81)	77 (26.74)	6.912	.009

*Note*: Data are expressed as the mean ± standard deviation (SD) or *N* (%).

Abbreviations: ACEI, angiotensin‐converting enzyme inhibitors; ARB, angiotensin receptor blocker; BMI, body mass index; BP, blood pressure; LAD, left anterior descending coronary; LCX, left circumflex branch; MACE, major adverse cardiac events; NLR, neutrophil‐to‐lymphocyte ratio; PLR, platelets to lymphocytes; RA, right coronary; WBC, white blood cells.

### 4.3 ROC curve analysis

**Figure 1:**
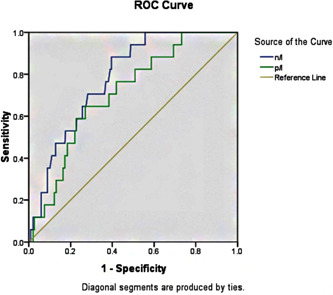
ROC analysis of PLR and NLR for 30‐day MACE. MACE, major adverse cardiac events; PLR, platelets to lymphocytes.

As illustrated in Figure 1, ROC analysis revealed that the PLR cutoff value was 178, with 65% sensitivity and 73% specificity for predicting 30‐day MACE (area [AUC] = 0.710, [*p* = .004]) (Figure 1). In comparison, the NLR threshold of 4.86 exhibited a recognition rate of 88% and a prediction accuracy of 61% (AUC = 0.783, *p* = .001). According to the correlation study, PLR shows a positive connection with NLR (*r* = .267, *p* = .001).

### 4.4 Multivariate analysis

**Figure 2:**
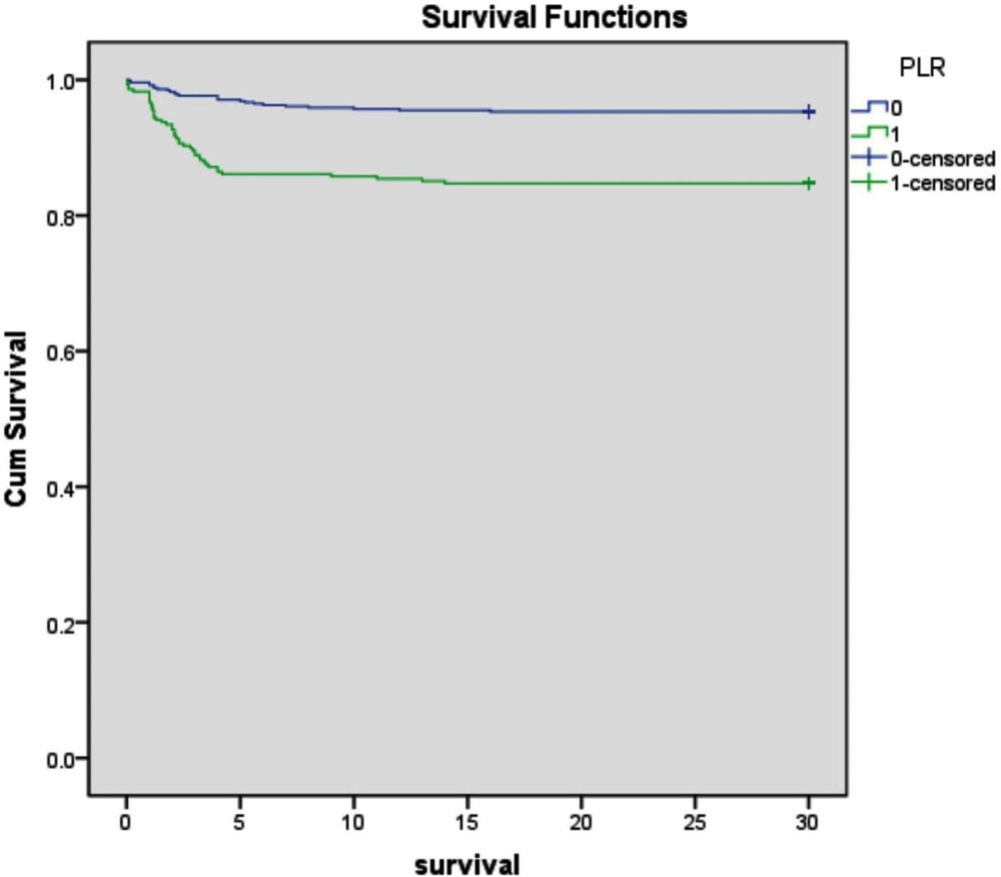
Kaplan−Meier curves for 30‐day MACE in patients with AMI. Survival for patients with PLR over and under 178. AMI, acute myocardial infarction; MACE, major adverse cardiac events; PLR, platelets to lymphocytes.

In the modified multivariate logistic model, NLR and PLR continue to correlate independently with 30‐day MACE (Table 3). For the purpose of analyzing “time‐to‐event” data, the KM approach is utilized. The all‐cause mortality rate is frequently included as the outcome in KM analyses; however, alternative outcomes, such as the occurrence of a cardiovascular event, could also be included. KM analysis, as seen in Figure 2, further corroborated this finding. Specifically, those with a PLR larger than 178 had a higher accumulative risk of 30‐day MACE (log rank, *p* .001). With the exception of the lymphocyte count, all the previously stated blood parameters, NLR and PLR, were more prevalent in the high‐PLR group. High PLR increases the likelihood of nonfatal myocardial reinfarction, cardiovascular death, and MACE. PLR was discovered to have a positive connection with NLR. After controlling for several factors, the 30‐day MACE and NLR remained independently related with the PLR.

**Table 3:** Independent predictors of 30‐day MACE of the study groups.

	*B*	*SE*	Sig	Exp (*B*)	*p* Value
NLR	1.073	0.446	0.016	2.925	.05
PLR	0.839	0.306	0.006	2.314	.03
Constant	2.420	0.641	0.000	0.089	.01

Abbreviations: MACE, major adverse cardiac events; PLR, platelets to lymphocytes.

## 5 DISCUSSION

Platelets are essential components of thrombosis and have been linked to the development and progression of coronary thrombosis.^21^ Platelets also play an essential part in the development of atherosclerosis.^22^ In AMI patients, an increase in platelet count is related with an increase in infarct size and worsening.^23^ More platelets at baseline are related with a worse outcome in AMI patients.^24^, ^25^ However, lymphocytes act as a controlled and static inflammatory pathway, which is suggestive of a repressed immune response.^26^ PLR provides an insight of pathways associated with aggregation and inflammation. It may serve as an indicator of thrombotic and inflammatory processes that cause heart damage.^27^ High PLR readings have been linked to severe coronary atherosclerosis in individuals with CAD and AMI, according to previous studies.^28^, ^29^, ^30^ According to Ayca et al. and Temiz et al., the PLR may predict the incidence rate of in‐hospital MACE in AMI patients.^31^, ^32^ Ugur et al. came to the conclusion that PLR has predictive significance in identifying and describing the short‐term clinical outcomes in STEMI.^33^, ^34^ In STEMI and NSTEMI populations, the link between PLR and long‐term prognosis has been verified twice.^27^, ^35^ In ACS patients, PLR has been identified as an independent predictor of systemic toxicity, including cardiac and all‐cause mortality, and myocardial infarction.^36^ Similar to earlier findings, the high PLR group is more likely to have nonfatal myocardial reinfarction, cardiovascular death, and MACE. ROC analysis revealed that the PLR cutoff value for predicting 30‐day MACE was 178, with a sensitivity of 65% and a specificity of 73%. The PLR cutoff value in the multivariate logistic model was likewise 178. In patients with a high PLR, the incidence of MACE was about 2.3 times higher than in individuals with a low PLR. Important in the etiology of atherogenesis and atherothrombosis are subtypes of WBC. After myocardial infarction, neutrophils may quickly penetrate the ischemic myocardium, triggering a damaging inflammatory response.^15^ In contrast, lymphocytes will be implicated in immune system control, and the advent of inflammation will enhance lymphocyte death.^37^ Neutrophilia might indicate inflammation, but a decrease in lymphocytes is the body's stress reaction. NLR is a more reliable predictor than a single measure because it incorporates the expected risks of neutrophil growth and lymphopenia.^38^, ^39^ Akpek et al. revealed that NLR may predict 30‐day death or poor prognosis independently in STEMI patients.^40^ Gul et al. revealed the prognostic usefulness of the NLR in ACS patients' 3‐year cardiovascular mortality.^41^ The NLR correlates substantially with 1‐year cardiovascular mortality in patients with STEMI or NSTEMI treated within 24 h with PCI. In the current research, a greater PLR will result in a greater NLR, confirming the positive link between PLR and NLR.^42^, ^43^ Multivariate logistic models examining independent correlations between NLR or PLR and 30‐day MACE were adjusted for the following factors. In patients with a high PLR, the incidence of MACE was about 2.8 times higher than in individuals with a low PLR. Patients with AMI may benefit from using PLR as a risk stratification and classification criterion because it is readily available, reliable, and cost effective.

## 6 LIMITATIONS

This research has two significant drawbacks. First, the data were not gathered from several units, the sample size was small, and patient selection bias may have impacted the results. A greater number of patients is required, particularly those with NSTEMI. Consider a suitable extension of the follow‐up time to better accurately anticipate patient risk.

## 7 CONCLUSION

PLR and NLR were linked with 30‐day MACE in AMI patients treated with primary PCI, according to the obtained data. These findings showed that the PLR might be regarded as a potentially accessible, dependable, and affordable criterion for risk stratification and categorization in AMI patient groups.

## AUTHOR CONTRIBUTIONS

**Hongling Wang**: Conceptualization. **Li Li**: Data curation. **Yi Ma**: Formal analysis and writing—reviewing and editing.

## CONFLICT OF INTEREST STATEMENT

The authors declare no conflict of interest.

## Data Availability

The data that support the findings of this study are available on request from the corresponding author. The data are not publicly available due to privacy or ethical restrictions.
